# A novel potent autophagy inhibitor ECDD-S27 targets vacuolar ATPase and inhibits cancer cell survival

**DOI:** 10.1038/s41598-019-45641-x

**Published:** 2019-06-24

**Authors:** Jiraporn Paha, Phongthon Kanjanasirirat, Bamroong Munyoo, Patoomratana Tuchinda, Naravut Suvannang, Chanin Nantasenamat, Kanokthip Boonyarattanakalin, Prasat Kittakoop, Sirawit Srikor, Gunganist Kongklad, Noppawan Rangkasenee, Suradej Hongeng, Pongsak Utaisincharoen, Suparerk Borwornpinyo, Marisa Ponpuak

**Affiliations:** 1 Department of Microbiology, Faculty of Science, Mahidol University, Bangkok, 10400 Thailand; 2 Excellent Center for Drug Discovery, Faculty of Science, Mahidol University, Bangkok, 10400 Thailand; 3 Department of Chemistry, Faculty of Science, Mahidol University, Bangkok, 10400 Thailand; 4 Center of Data Mining and Biomedical Informatics, Faculty of Medical Technology, Mahidol University, Bangkok, 10700 Thailand; 5 College of Nanotechnology, King Mongkut’s Institute of Technology Ladkrabang, Bangkok, 10520 Thailand; 6 Chulabhorn Research Institute, Bangkok, 10210 Thailand; 7 Chulabhorn Graduate Institute, Chemical Biology Program, Bangkok, 10210 Thailand; 8 Center of Excellence on Environmental Health and Toxicology, CHE, Ministry of Education, Bangkok, Thailand; 9 Department of Physics, Faculty of Science, Mahidol University, Bangkok, 10400 Thailand; 10 Department of Pediatrics, Faculty of Medicine Ramathibodi Hospital, Mahidol University, Bangkok, 10400 Thailand; 11 Department of Biotechnology, Faculty of Science, Mahidol University, Bangkok, 10400 Thailand

**Keywords:** Macroautophagy, Molecular medicine

## Abstract

Autophagy is a conserved lysosomal-dependent cellular degradation process and its dysregulation has been linked to numerous diseases including neurodegeneration, infectious diseases, and cancer. Modulation of autophagy is therefore considered as an attractive target for disease intervention. We carried out a high-content image analysis screen of natural product-derived compounds to discover novel autophagy modulating molecules. Our screen identified ECDD-S27 as the most effective compound for increasing the number of autophagic vacuoles inside cells. The structure of ECDD-S27 revealed that it is a derivative of cleistanthin A, a natural arylnaphthalene lignan glycoside found in plants. ECDD-S27 increases the number of autophagic vacuoles by inhibiting the autophagic flux and is able to restrict the survival of different cancer cells at low nanomolar concentrations. Molecular docking and SERS analysis showed that ECDD-S27 may potentially target the V-ATPase. Upon treatment of various cancer cells with ECDD-S27, the V-ATPase activity is potently inhibited thereby resulting in the loss of lysosomal acidification. Taken together, these data indicated that ECDD-S27 retards the autophagy pathway by targeting the V-ATPase and inhibits cancer cell survival. The observed antitumor activity without cytotoxicity to normal cells suggests the therapeutic potential warranting further studies on lead optimization of the compound for cancer treatment.

## Introduction

Autophagy is an evolutionarily conserved homeostatic degradation process of cytoplasmic substances that normally occurs inside eukaryotic cells for self-cleansing and can be upregulated during time of stresses^1,2^. Autophagy can be induced by various conditions such as starvation, drug exposure, and immune mediators as well as be inhibited by numerous compounds^3,4^. During autophagy, the double-membrane autophagosomes engulf the cytosolic substrates and deliver them to lysosomes for digestion^5^. These substrates include defective macromolecules, defunct organelles, and even invading whole pathogens^6^. Autophagosomes are formed through the co-operative work of various autophagy-related (ATG) proteins organized into complexes which include the initiating complex ULK1/2 protein kinase, the nucleation complex Beclin 1/PI3K/ATG14, and the elongation complex ubiquitin-like conjugating systems ATG12-ATG5-ATG16 and LC3^2,5,7^. Upon closure of the autophagosomes, the Beclin 1/PI3P/UVRAG complex then promotes the fusion of autophagosomes with the acidic lysosomes^8^, resulting in the delivery of lysosomal hydrolases to degrade the engulfed contents. For the past decade, autophagy has been increasingly appreciated for its role in various diseases including neurodegeneration^9^, aging^10^, inflammation^11,12^, infectious diseases^4^, and cancer^13–16^. Therefore, manipulation of autophagy pathway holds a great promise for new therapeutic applications.

In the case of cancer, autophagy has been shown to play two opposing roles. On one hand, autophagy functions as a tumor suppressor mechanism in which it facilitates the clearance of damaged macromolecules and organelles thereby preventing excessive ROS production and genome damage and thus preventing normal cells to become cancerous^17^. However, once the cells become malignant, autophagy plays a pro-survival role in protecting cancer cells from metabolic and therapeutic stresses thereby promoting the growth of the established tumors^17^. Extensive data showed that autophagy is upregulated in different cancer types and autophagy impairment by genetic or pharmaceutical inhibition can limit cancer cell survival, dissemination, and metastasis in *in vitro* studies and *in vivo* animal tumor models^17^. Therefore, inhibiting autophagy is currently being developed as a new strategy for cancer treatment. Multiple clinical trials on autophagy inhibitors which include chloroquine (CQ) and its derivative hydroxychloroquine (HCQ) either alone or in combination with other cancer drugs or radiation are now being conducted in a wide range of tumors and the results demonstrated some improving clinical outcomes for cancer patients^14,15^. Both CQ and HCQ block acidification of the lysosomes and thereby inhibiting the autophagosome-lysosome fusion and hence the autophagic flux^18^. As high micromolar concentrations of CQ and HCQ are required to inhibit autophagy which may limit their clinical use^19–23^, the continued search for more potent autophagy inhibitors is warranted. Taken together, these findings supported the idea and potential use of autophagy inhibitors for anticancer therapy.

As mentioned above, impaired autophagy has been implicated in different pathophysiological conditions and modulation of autophagy is viewed as an attractive new strategy for disease treatment. In this work, we set out to identify autophagy modulating molecules from natural product-derived compounds by using the fluorescently-based high-content (HC) image screen. From the screen, ECDD-S27 was identified as the compound that potently increases the number of autophagic vacuoles in cells. Further characterization on ECDD-S27 mechanism of action revealed that it is an autophagic flux inhibitor that can strongly restrict the viability of different cancer cell types while not toxic to normal cells. Our molecular docking, SERS, and functional analyses identified vacuolar ATPase as the target of ECDD-S27. The lack of synergistic effect between bafilomycin A1, a well-known autophagic flux inhibitor, and ECDD-S27 in cancer cell restriction further supported the involvement of ECDD-S27 in targeting this pathway and thereby inhibits the survival of cancer cells. These data indicated the potential development of ECDD-S27 as a lead compound for cancer.

## Results

### Identification of natural product-derived autophagy modulating compounds

**Figure 1:**
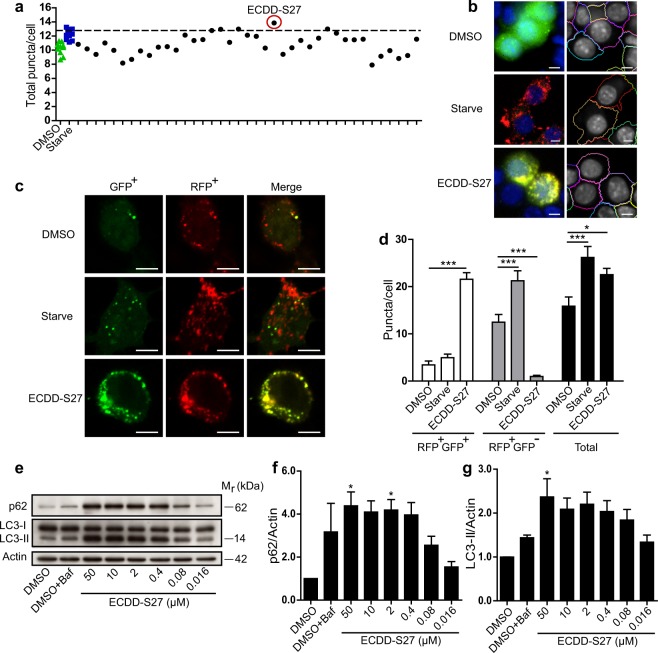
ECDD-S27 is a potent autophagic flux inhibitor. (**a,b**) Screening of natural product-derived compounds for their autophagy modulating activity. Raw264.7 macrophages were transfected with cDNAs encoding RFP-GFP-LC3B. At 48 h post transfection, cells were treated with DMSO (negative control), starvation (positive control), or natural product-derived compounds (50 µM) for 4 h. Cells were then fixed and analyzed by HC image analysis to quantify the number of total LC3B puncta per cell. The dashed line represents 3 S.D. above that of the mean of the DMSO treated control. ECDD-S27 was identified as the most effective compound to increase the number of total LC3B puncta per cell from the screen. Representative images of the HC image analysis with boundary of cells (right panels). Bar 5 µm. (**c,d**) ECDD-S27 inhibits autophagic flux. Raw264.7 macrophages expressing RFP-GFP-LC3B were treated with DMSO, starvation, or 50 µM of ECDD-S27 for 4 h. Cells were then processed for confocal microscopy analysis and the number of RFP^+^GFP^+^-LC3B (autophagosomes) and RFP^+^GFP^−^-LC3B (autolysosomes) puncta per cell was quantified. Only puncta ≥0.3 µm in size were counted. Data are the means ± SEM from at least three independent experiments. At least 30 cells per condition per independent experiment were quantified; *p < 0.05 and ***p < 0.001, all relative to the DMSO control, were determined by one-way ANOVA with a Tukey’s multiple comparison test. Bar 5 µm. (**e**–**g**) LC3-II and p62 immunoblots confirmed inhibition of autophagic flux by ECDD-S27. Raw264.7 macrophages were treated with DMSO with or without bafilomycin A1 or ECDD-S27 at the indicated concentrations for 4 h. Representative images cropped from the same blot are shown and full images are included in the supplementary information. The intensities of LC3-II, p62, and Actin were quantified using ImageJ. The graphs showed densitometric analysis of p62/Actin and LC3-II/Actin levels. The IC_50_ value of ECDD-S27 in inhibiting autophagic flux was ≤ 0.016 µM. Data are mean ± SEM; *p < 0.05, all relative to the DMSO control from three independent experiments, was determined by one-way ANOVA with a Tukey’s multiple comparison test.

As defective autophagy has been linked to a number of medical conditions, several drug discovery screens of small compound libraries and FDA-approved drugs have been conducted to identify autophagy modulating compounds^24–30^. Since Thai herbal and natural product-based traditional medicines have been used as therapeutics for diseases, we are interested to see whether autophagy modulating activity could be found in these molecules and their derivatives. We therefore conducted the HC imaging screen by quantitating the number of LC3B puncta, the biological marker for autophagic vacuoles in cells, upon treatment with the Thai natural product-derived compounds deposited into the Excellent Center for Drug Discovery, Mahidol University. In brief, RAW264.7 macrophages expressing mRFP-GFP-LC3B were treated with DMSO (negative control), starvation (positive control), or 50 µM of each compound for 4 h and processed for HC image analysis. The number of total autophagosomes (RFP^+^GFP^+^-LC3B) and autolysosomes (RFP^+^GFP^−^LC3B) per cell was then quantified. ECDD-S27 was identified as the most effective compound in increasing the number of total LC3B puncta per cell from our screen (Fig. 1a,b).

### ECDD-S27 potently inhibits the autophagic flux

Since an increased number of LC3B puncta per cell by a compound could be resulted from either autophagy induction or autophagic flux inhibition activity^31^, we performed a more careful analysis of the RFP^+^GFP^+^-LC3B versus RFP^+^GFP^–^LC3B number per cell using confocal microscopy in order to determine the ECDD-S27 mode of action. The results showed that ECDD-S27 increases the number of autophagosomes but decreases the number of autolysosomes per cell when compared to that of the DMSO treated control, indicating that it acts by inhibiting the autophagic flux (Fig. 1c,d). This is in contrast to the results seen in cells undergoing starvation (autophagy induction control), in which both the number of autophagosomes and autolysosomes are increased when compared to that of the DMSO treated cells (Fig. 1c,d). In addition, to confirm that ECDD-S27 works by inhibiting the autophagic flux, p62 and lipidated LC3 (LC3-II) protein levels were examined by immunoblotting. Both p62 and LC3-II are substrates for autophagic degradation in the autolysosomes and hence their elevated levels are an indication of autophagic flux retardation^31^. When cells were treated with varied concentrations of ECDD-S27, the levels of p62 and LC3-II are increased in a dose dependent manner, confirming that ECDD-S27 functions by hampering the autophagic flux (Fig. 1e–g). ECDD-S27 strongly blocks the autophagic flux at low nanomolar concentrations when compared to that of 100 nM bafilomycin A1 used as the standard autophagic flux inhibitor control (Fig. 1e–g). Thus, ECDD-S27 is a novel potent natural product-derived autophagic flux inhibitor.

### ECDD-S27 is a derivative of cleistanthin A

**Figure 2:**
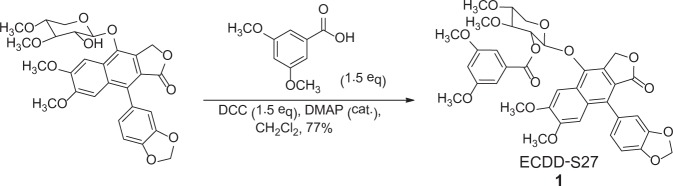
Chemical structure of ECDD-S27. The newly identified potent natural-product derived autophagic flux inhibitor, ECDD-S27, is a derivative of cleistanthin A, a natural arylnaphthalene lignan glycoside found in *Phyllanthus taxodiifolius* Beille (Euphorbiaceae). ECDD-S27 is derived from the esterification of cleistanthin A with 3,5-dimethoxybenzoic acid, in the presence of *N,N*′-dicyclohexylcarbodiimide (DCC) and a catalytic amount of 4-dimethylaminopyridine (DMAP), resulting in the formation of a new ester, 2-(9-(1,3-benzodioxol-5-yl)-6,7-dimethoxy-1-oxo-1,3-dihydrobenzo[f][2]benzofuran-4-yl)oxy-4,5-dimethoxytetrahydro-*2H*-pyran-3-yl 3,5-dimethoxybenzoate (ECDD-S27, 1).

Investigation of ECDD-S27 structure revealed that it is a derivative of cleistanthin A, a well-known natural arylnaphthalene lignan glycoside found in plants *Phyllanthus taxodiifolius* Beille (Euphorbiaceae) and *Cleistanthus collinus*^32–36^. ECDD-S27 was derived from the esterification of cleistanthin A with 3,5-dimethoxybenzoic acid in the presence of *N,N*′-dicyclohexylcarbodiimide (DCC) and a catalytic amount of 4-dimethylaminopyridine (DMAP), resulting in the formation of a new ester, 2-(9-(1,3-benzodioxol-5-yl)-6,7-dimethoxy-1-oxo-1,3-dihydrobenzo[f][2]benzofuran-4-yl)oxy-4,5-dimethoxytetrahydro-*2H*-pyran-3-yl 3,5-dimethoxybenzoate (**ECDD-S27, 1**) (Fig. 2). The structure of ECDD-S27 was confirmed by spectroscopic methods (^1^H-NMR, ^13^C-NMR, UV, IR and MS analysis) (Please see Methods for more information). Purity of ECDD-S27 was determined to be 97% by using the HPLC technique (column C_18_ shiseido, 80% MeOH/H_2_O, 1 mL/min). Note that while ECDD-S27 was identified as a positive compound from our screen that can effectively increase the number of total LC3B puncta per cell (Fig. 1a,b), the parent compound cleistanthin A does not increase the number of LC3B puncta and hence not a positive compound in our screen.

### ECDD-S27 restrains autophagic flux and decreases colorectal adenocarcinoma HT-29 cell viability

**Figure 3:**
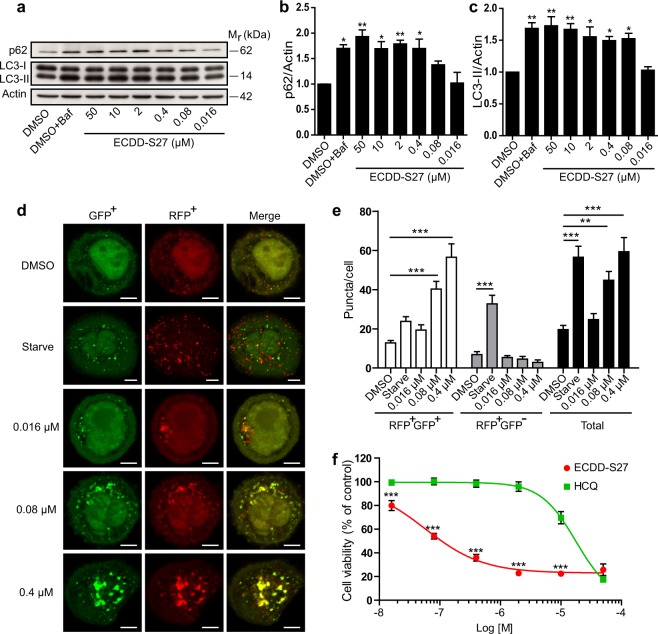
ECDD-S27 potently inhibits autophagic flux and restricts colorectal adenocarcinoma HT-29 cell survival. (**a–c)** Autophagic flux analysis in HT-29 cells by LC3-II and p62 immunoblots. Cells were treated with DMSO with or without bafilomycin A1 or ECDD-S27 at the indicated concentrations for 4 h. Representative images cropped from the same blot are shown and full images are included in the supplementary information. The intensities of LC3-II, p62, and Actin were quantified using ImageJ. The graphs showed densitometric analysis of p62/Actin and LC3-II/Actin expression levels. ECDD-S27 inhibits autophagic flux with the IC_50_ value of ≤0.016 µM. Data are mean ± SEM; *p < 0.05 and **p < 0.01, all relative to the DMSO control from three independent experiments, were determined by one-way ANOVA with a Tukey’s multiple comparison test. **(d–e**) RFP-GFP-LC3B puncta analysis confirmed ECDD-S27 autophagic flux inhibition. RFP-GFP-LC3B expressing HT-29 cells were treated with DMSO, starvation, or ECDD-S27 at the indicated concentrations for 4 h and processed for confocal microscopy. The number of LC3B puncta/cell was then analyzed. Only puncta ≥0.3 µm in size were counted. Data are the means ± SEM from at least three independent experiments. At least 30 cells per condition per independent experiment were quantified; **p < 0.01 and ***p < 0.001, all relative to the DMSO control, were determined by one-way ANOVA with a Tukey’s multiple comparison test. Bar 5 µm. **(f)** ECDD-S27 inhibits HT-29 cell survival. Cells were treated with DMSO (negative control), ECDD-S27, or HCQ at the indicated concentrations for 72 h and their viability was measured by the MTS assay. Data are mean ± SD from three independent experiments; the results were expressed relative to the DMSO control, defined as 100%. ***p < 0.001, ECDD-S27 vs HCQ treatment at the same concentration, was determined by one-way ANOVA with a Tukey’s multiple comparison test.

As numerous tumor cells were found to depend on autophagy for their survival and autophagic flux inhibitors are currently being tested in clinical trials against cancers^13–15^, we set out to examine the effect of ECDD-S27 on different cancer cells. To determine this, we first treated the colorectal adenocarcinoma HT-29 cells with various concentrations of ECDD-S27 and measured the alteration in autophagic flux by p62 and LC3-II immunoblotting. ECDD-S27 is able to increase the levels of p62 and LC3-II in a dose dependent manner with the IC_50_ value of ≤0.016 µM (Fig. 3a–c). In addition, we further confirmed the autophagic flux inhibition activity of ECDD-S27 in these cells by using the LC3B puncta assay. When mRFP-GFP-LC3B expressing HT-29 cells were treated with starvation to induce the autophagic flux, the enhanced number of both autophagosomes and autolysosomes was observed (Fig. 3d,e). In contrast, when cells were treated with low nanomolar concentrations of ECDD-S27, only the number of autophagosomes is increased while the number of autolysosomes is slightly decreased (Fig. 3d,e), confirming the autophagic flux retardation activity of ECDD-S27. To assess the ability of ECDD-S27 in restricting HT-29 cell survival, we treated the cells with different concentrations of ECDD-S27 and determine the cell viability using the MTS assay. ECDD-S27 is able to potently limit HT-29 cell survival with the IC_50_ value of 0.06 µM (Fig. 3f). The activity of ECDD-S27 in decreasing the HT-29 cell survival is better than that of HCQ (IC_50_ = 18.4 µM) (Fig. 3f). Thus, ECDD-S27 is able to strongly inhibit the autophagic flux and HT-29 cell viability at low nanomolar concentrations.

### Autophagic flux and survival of hepatocellular carcinoma HepG2 cells are blocked by ECDD-S27

**Figure 4:**
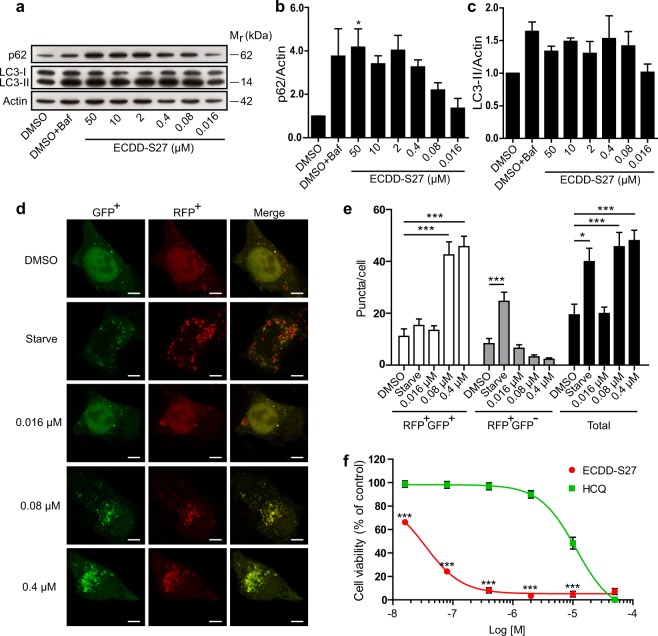
Autophagic flux arrest and survival inhibition by ECDD-S27 in hepatocellular carcinoma HepG2 cells. (**a–c)** LC3-II and p62 Western blot analysis to assess autophagic flux in HepG2 cells. Cells were treated with DMSO with or without bafilomycin A1 or ECDD-S27 at the indicated concentrations for 4 h. Representative images cropped from the same blot are shown and full images are included in the supplementary information. The intensities of LC3-II, p62, and Actin were quantified using ImageJ. p62/Actin and LC3-II/Actin levels were then determined. The IC_50_ value of ECDD-S27 for autophagic flux inhibition was 0.016–0.080 µM. Data are mean ± SEM; *p < 0.05, all relative to the DMSO control from three independent experiments, was determined by one-way ANOVA with a Tukey’s multiple comparison test. **(d–e**) Confirmation of ECDD-S27 autophagic flux inhibition by RFP-GFP-LC3B puncta assay. HT-29 cells expressing RFP-GFP-LC3B were treated with DMSO, starvation, or ECDD-S27 at the indicated concentrations for 4 h, processed for confocal microscopy, and analyzed for the number of LC3B puncta/cell. Only puncta ≥0.3 µm in size were counted. Data are the means ± SEM from at least three independent experiments. At least 30 cells per condition per independent experiment were quantified; *p < 0.05 and ***p < 0.001, all relative to the DMSO control, were determined by one-way ANOVA with a Tukey’s multiple comparison test. Bar 5 µm. **(f)** ECDD-S27 decreases HepG2 cell survival. Cells were treated with DMSO (negative control), ECDD-S27, or HCQ at the indicated concentrations for 72 h and their viability was measured by the MTS assay. Data are mean ± SD from three independent experiments; the results were expressed relative to the DMSO control, defined as 100%. ***p < 0.001, ECDD-S27 vs HCQ treatment at the same concentration, was determined by one-way ANOVA with a Tukey’s multiple comparison test.

To investigate whether ECDD-S27 can arrest autophagic flux in other cancer cell types, we performed the Western blot analysis of p62 and LC3-II levels in the hepatocellular carcinoma HepG2 cells upon treatment with different concentrations of ECDD-S27. The elevated p62 and LC3-II protein levels were observed starting at low nanomolar concentrations of ECDD-S27 and increased in a dose dependent manner (IC_50_ = 0.016–0.080 µM) (Fig. 4a–c). In addition, the mRFP-GFP-LC3B puncta assay was conducted and ECDD-S27 can effectively increase the number of autophagosomes but not that of autolysosomes, demonstrating the autophagic flux inhibition activity of ECDD-S27 in these cells (Fig. 4d,e). Moreover, ECDD-S27 can strongly reduce HepG2 cell survival as determined by the MTS assay (IC_50_ = 0.03 µM) and its activity is more potent than that observed when cells were treated with HCQ (IC_50_ = 11.44 µM) (Fig. 4f). These results confirmed that ECDD-S27 can limit the autophagic flux and cell viability of the hepatocellular carcinoma HepG2 cells.

### ECDD-S27 inhibits autophagic flux and survival of cervical adenocarcinoma HeLa cells

**Figure 5:**
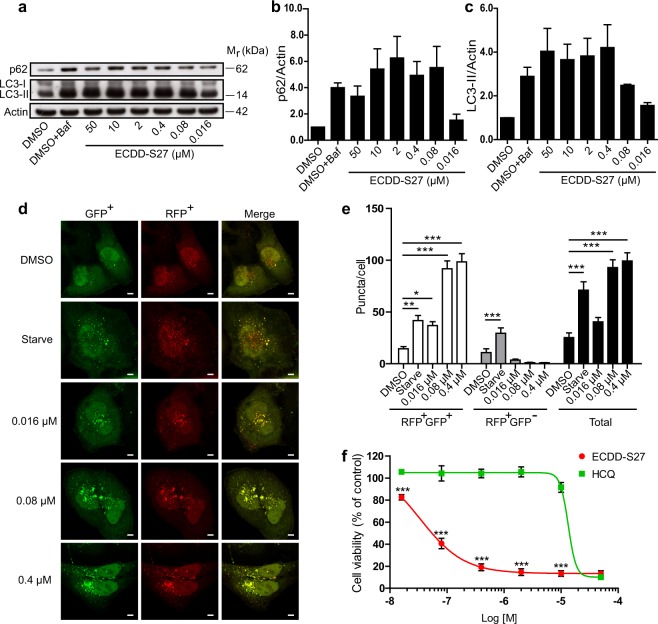
Decrease in autophagic flux and cell survival in cervical adenocarcinoma HeLa cells by ECDD-S27. (**a–c**) ECDD-S27 inhibits autophagic flux in HeLa cells. Cells were treated with DMSO with or without bafilomycin A1 or ECDD-S27 at the indicated concentrations for 4 h. The expression levels of p62/Actin and LC3-II/Actin were then assessed using immunoblotting followed by the densitometric analysis using ImageJ. Representative images cropped from the same blot are shown and full images are included in the supplementary information. ECDD-S27 inhibits autophagic flux with the IC_50_ of 0.016–0.080 µM. Data are mean ± SEM from three independent experiments. **(d–e**) ECDD-S27 autophagic flux inhibition in HeLa cells was confirmed by RFP-GFP-LC3B puncta assay. RFP-GFP-LC3B expressing HeLa cells were treated with DMSO, starvation, or ECDD-S27 at the indicated concentrations for 4 h and analyzed by confocal microscopy. The number of LC3B puncta/cell was then quantified. Only puncta ≥0.3 µm in size were counted. Data are the means ± SEM from at least three independent experiments. At least 30 cells per condition per independent experiment were quantified; *p < 0.05, **p < 0.01, and ***p < 0.001, all relative to the DMSO control, were determined by one-way ANOVA with a Tukey’s multiple comparison test. Bar 5 µm. **(f)** HeLa cell survival is restricted by ECDD-S27. Cells were treated with DMSO (negative control), ECDD-S27, or HCQ at the indicated concentrations for 72 h and their viability was measured by the MTS assay. Data are mean ± SD from three independent experiments; the results were expressed relative to the DMSO control, defined as 100%. ***p < 0.001, ECDD-S27 vs HCQ treatment at the same concentration, was determined by one-way ANOVA with a Tukey’s multiple comparison test.

We also examined the activity of ECDD-S27 in the cervical adenocarcinoma HeLa cells. Immunoblot analysis showed a dose dependent enhancement levels of p62 and LC3-II in these cells upon treatment with varied concentrations of ECDD-S27 (IC_50_ = 0.016–0.080 µM) (Fig. 5a–c). In addition, the augmented number of autophagosomes but not that of autolysosomes was observed in mRFP-GFP-LC3B expressing ECDD-S27-treated HeLa cells, thus confirming the blockage of autophagic flux by ECDD-S27 (Fig. 5d,e). Furthermore, the viability of HeLa cells was greatly suppressed by ECDD-S27 (IC_50_ = 0.04 µM) when compared to that of the HCQ treated cells (IC_50_ = 13.56 µM) (Fig. 5f). Altogether, these data demonstrated that ECDD-S27 is a potent inhibitor of the autophagic flux and able to restrict cell survival of different cancer cell types. To also assess whether ECDD-S27 is toxic to normal cells, we performed the toxicity test using the MTT assay upon treatment of the human kidney normal cell HK-2 cells with varied concentrations of ECDD-S27. The results showed that ECDD-S27 is nontoxic to HK-2 cells (IC_50_ > 50 µM) (Supplementary Fig. S1).

### ECDD-S27 suppresses autophagic flux by targeting the vacuolar ATPase

**Figure 6:**
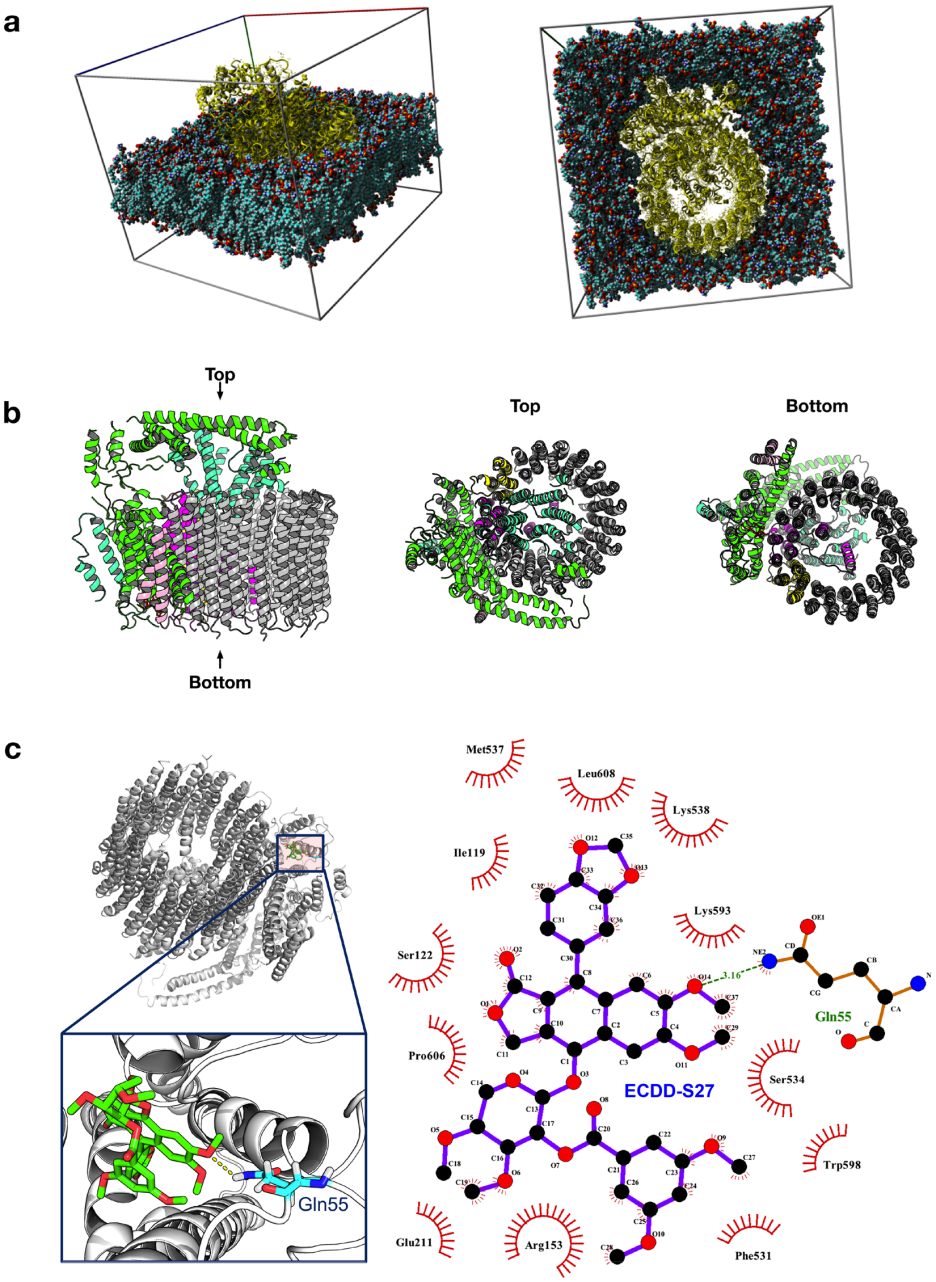
Molecular simulation for elucidating the potential binding between ECDD-S27 and V-ATPase. (**a**) Molecular dynamics simulation of the protein-lipid membrane system. (**b)** Side, top, and bottom views of the V-ATPase protein structure. **(c)** Left panel illustrates the location and zoom-up view of the binding pocket showing the hydrogen bond interaction between the ether oxygen atom from ECDD-S27 with the amide nitrogen atom from Gln55. Right panel shows the two-dimensional schematic diagram of the protein-ligand interaction.

To investigate the molecular mechanism of ECDD-S27 in autophagic flux arrest, we looked at the substructural components of ECDD-S27 (Fig. 2). The core structure of ECDD-S27 consists of diphyllin [1-hydroxy-2-(hydroxymethyl)-6,7-dimethoxy-4-(3,4-methylenedioxy-phenol)-3-naphthoic acid-γ-lactone], which has previously been shown to inhibit the vacuolar ATPase (V-ATPase) activity and thereby the lysosomal acidification in osteoclasts and cancer cells^37–39^. Thus, it is plausible that ECDD-S27 may block the V-ATPase activity and thereby retarding the autophagic flux in our cells. In order to determine whether ECDD-27 could potentially target the V-ATPase, we performed a protein-ligand interaction study that constitutes an initial molecular dynamics simulation of the protein structure in a lipid bilayer water box followed by molecular docking. Representative docking pose from the highest populated cluster having the lowest energy was selected for post-docking analysis. As shown in Fig. 6a, ECDD-S27 binds to the eukaryotic V-ATPase with a binding energy of −7.8 kcal/mol. Hydrogen bond interaction is formed between the ether oxygen atom from ECDD-S27 with the amide nitrogen atom of Gln55 on subunit e along with hydrophobic interaction formed with Ser122, Leu119, and Glu211 on subunit c″, Arg153 on subunit c, and Phe531, Ser534, Met537, Lys538, Trp598, Pro606 and Leu608 on subunit a (Fig. 6b,c).

**Figure 7:**
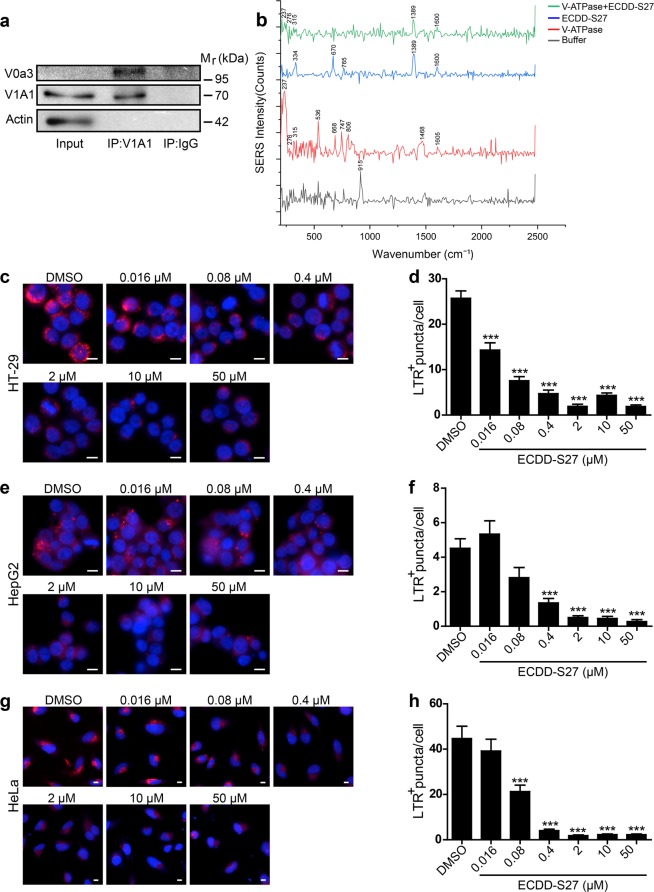
ECDD-S27 targets the vacuolar ATPase. (**a)** Isolation of human V-ATPase by immunoprecipitation. HeLa cells were lysed in RIPA buffer supplemented with protease inhibitors. Anti-V1A1 antibody was used to isolate the V-ATPase from the lysate using the normal rabbit IgG antibody as the negative control. The precipitated immune complex was then analyzed by Western blot analysis for the presence of V1A1 and V0a3, markers for V_1_ and V_0_ domains of the V-ATPase, respectively. **(b)** SERS analysis of the ECDD-S27 and V-ATPase interaction. The isolated human V-ATPase was treated with 5 mM ECDD-S27 at 4 °C overnight followed by washing three times with PBS containing 0.5% NP-40 to wash out the unbound compound. The sample was then subjected to SERS analysis. The V-ATPase alone, ECDD-S27 alone, and buffer alone were used as controls. HT-29 **(c,d**), HepG2 **(e–f)**, and HeLa **(g,h)** cells were treated with DMSO or ECDD-S27 at the indicated concentrations for 2 h. Acidification of lysosomes was then determined by addition of the Lysotracker Red (LTR) dye to these cells in the presence of DMSO or ECDD-S27 and incubation was continued for 2 h. Cells were then fixed and processed for HC image analysis. The number of LTR^+^ puncta per cell was then quantified. Bar 10 µm. Data are shown as mean ± SEM; ***p < 0.001, all relative to the DMSO control, was determined by one-way ANOVA with a Tukey’s multiple comparison test.

In addition to the molecular docking results described above, we further examined the interaction of ECDD-S27 with V-ATPase by conducting surface-enhanced Raman substrates (SERS) analysis. Firstly, the human V-ATPase was isolated from HeLa cells by immunoprecipitation using the anti-V1A1 antibody. Western blot analysis of the precipitated immune complex showed that the anti-V1A1 antibody can isolate V1A1 and V0a3 proteins, both of which are components of the V_1_ and V_0_ domains of the V-ATPase respectively, from the HeLa cell lysate while the normal rabbit IgG antibody that was used as the negative control could not (Fig. 7a). This indicated that our immunoprecipitation can successfully isolate the endogeneous human V-ATPase protein complex from HeLa cells. Afterwards, the interaction between the isolated human V-ATPase protein complex and ECDD-S27 was examined by SERS analysis. This was conducted by treating the isolated human V-ATPase with 5 mM ECDD-S27 at 4 °C overnight followed by multiple washes in PBS containing 0.5% NP-40 as to remove the unbound compound. Then, the V-ATPase alone, the ECDD-S27 alone, and the ECDD-S27 treated V-ATPase samples were subjected to SERS analysis. Figure 7b showed SERS spectrum of the V-ATPase, ECDD-S27, and ECDD-S27 treated V-ATPase. The Raman pattern of ECDD-S27 is composed of peaks at 1389 and 1600 cm^−1^ as observed as high intensity peaks in the ECDD-S27 alone sample in addition to the lower intensity peaks at 334, 670, and 765 cm^−1^. Interestingly, in the ECDD-S27 treated V-ATPase sample, the ECDD-S27 characteristic peaks at 1389 and 1600 cm^−1^ as well as peaks at 237, 276, 315 cm^−1^ corresponding to those identified in the V-ATPase alone sample can be observed. The weakening of the ECDD-S27 characteristic peaks at 1389 and 1600 cm^−1^ in concurrent with the disappearance of peaks at 334, 670, and 765 cm^−1^ in the ECDD-S27 treated V-ATPase sample suggested that ECDD-S27 could directly interact with the V-ATPase. In addition, no new peaks were observed in the ECDD-S27 treated V-ATPase sample, thereby confirming our molecular docking results which showed no new covalent bond formed between ECDD-S27 and the V-ATPase.

Moreover, to functionally verify that ECDD-S27 can inhibit the V-ATPase activity, we treated HT-29, HepG2, and HeLa cells with varied concentrations of ECDD-S27 and measured the lysosomal acidification by staining the acidic organelles with the LysoTracker Red (LTR) dye. The HC image analysis was then used to quantify the number of LTR^+^ vacuoles per cell. The results showed that ECDD-S27 can markedly decrease the number of acidified organelles in HT-29 (Fig. 7c,d), HepG2 (Fig. 7e,f), and HeLa (Fig. 7g,h) cells in a dose dependent manner when compared to that of the DMSO treated control cells. Thus, these data supported that ECDD-S27 targets the V-ATPase and thereby deacidifies the lysosomes resulting in impaired autophagic flux.

### Causal relationship between autophagic flux inhibition by ECDD-S27 and cancer cell inhibition

**Figure 8:**
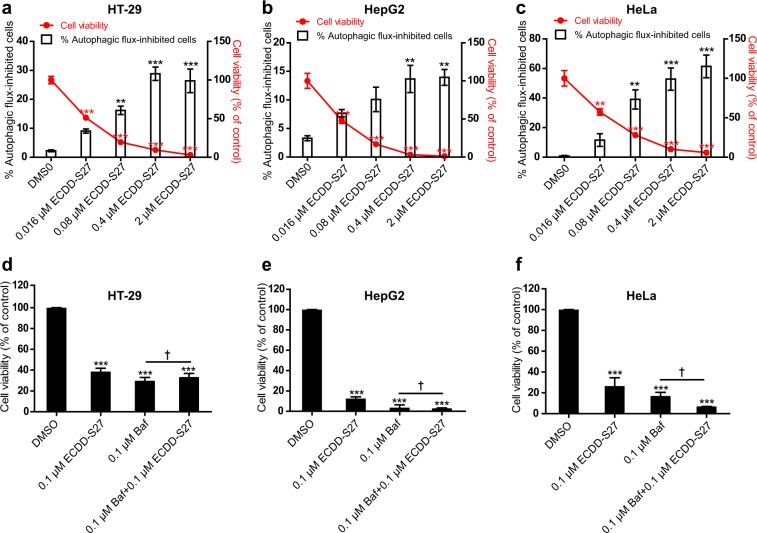
ECDD-S27 inhibits autophagic flux and decreases cancer cell survival. HT-29 (**a**), HepG2 (**b**), and HeLa (**c**) cells were transfected with cDNAs encoding GFP-LC3B. At 48 h after transfection, cells were treated with DMSO (negative control) or ECDD-S27 at indicated concentrations for 72 h. Cells were then stained with Hoechst and processed for HC image analysis. Percent cell viability and autophagic flux-inhibited cells were then analyzed. Data are shown as mean ± SEM. **p < 0.01 and ***p < 0.001, all relative to the DMSO control, were determined by one-way ANOVA with a Tukey’s multiple comparison test. HT-29 (**d**), HepG2 **(e)**, HeLa **(f)** cells were treated with DMSO (negative control), ECDD-S27, bafilomycin A1, or bafilomycin A1 in combination with ECDD-S27 at the indicated concentrations for 72 h and their viability was measured by the MTS assay. Data are shown as mean ± SD from at least two independent experiments; results were expressed relative to the DMSO control, defined as 100%. ^†^p ≥ 0.05 and ***p < 0.001, all relative to the DMSO control, were determined by one-way ANOVA with a Tukey’s multiple comparison test.

To investigate the causal relationship between autophagic flux inhibition by ECDD-S27 and cancer cell inhibition, we first measured whether autophagic flux inhibition by ECDD-S27 is correlated with cancer cell inhibition in GFP-LC3B expressing HT-29, HepG2, and HeLa cells treated with different concentrations of ECDD-S27 by HC image analysis. An increase in the number of GFP^+^-LC3B puncta (autophagosomes) in cells is used as a marker for autophagic flux inhibition. Results showed a dose-dependent increase in autophagic flux inhibition of GFP-LC3B expressing HT-29 (Fig. 8a), HepG2 (Fig. 8b), and HeLa (Fig. 8c) cells while there is a dose-dependent decrease in the survival of these cells upon treatment with increasing concentration of ECDD-S27. These data thus confirmed the correlation between autophagic flux inhibition by ECDD-S27 and cancer cell inhibition. To further examine the aforementioned relationship, we also treated HT-29, HepG2, and HeLa cells with bafilomycin A1, a standard autophagic flux inhibitor, and measured the cancer cell viability by means of the MTS assay. Upon treatment with the standard autophagic flux inhibitor bafilomycin A1, the survival of HT-29 (Fig. 8d), HepG2 (Fig. 8e), and HeLa (Fig. 8f) cells were found to be greatly inhibited at a similar level to that of ECDD-S27 treatment, thereby confirming that autophagic flux inhibition can result in cancer cell inhibition. In addition, the co-treatment of bafilomycin A1 with ECDD-S27 did not result in a further increase in cancer cell inhibition in these cells, thereby indicating that ECDD-S27 and bafilomycin A1 target the same pathway. Altogether, these data strongly suggested that autophagic flux inhibition by ECDD-S27 resulted in subsequent cancer cell inhibition.

In summary, our study showed that ECDD-S27 is a novel potent natural product-derived autophagic flux inhibitor that can restrict cancer cell viability at low nanomolar concentrations. It does so by targeting the V-ATPase resulting in the decrease in lysosomal acidification and thereby hampering the autophagosome-lysosome fusion. The ability of ECDD-S27 to potently decrease the viability of cancer cells while sparing normal cells indicated a great promise of this compound as a lead anti-cancer molecule.

## Discussion

Due to the involvement of autophagy in several pathophysiological conditions, the search for novel autophagy modulating small molecules is of high interest^40–44^. As many herbal and natural product-derived compounds have been used in traditional medicines to treat diseases, they hold a great pharmacological potential, though the underlying mechanisms possessed by these compounds are largely uncharacterized. The aim of this study was to identify natural product-derived compounds that exhibit autophagy modulating activity by using a phenotypic-based screening. Our high-content image analysis screen identified ECDD-S27, a derivative of cleistanthin A found in plants^32–36^, to potently increase the number of autophagic vacuoles in cells. Upon further characterization into its mechanism of action, we discovered that ECDD-S27 does so by targeting the V-ATPase and hence deacidifies the lysosomes leading to the block in autophagosome-lysosome fusion and thereby inhibiting the autophagic flux. As autophagic flux inhibitors, CQ and HCQ, are currently investigated in clinical trials either alone or in combination with chemotherapy and radiotherapy for cancers^13–15^, we tested the ability of ECDD-S27 in suppressing the viability of cancer cells. Our results conducted in different cancer cell types showed that ECDD-S27 can potently restrict cancer cell survival at low nanomolar concentrations while it is not toxic to normal cells, establishing the therapeutic potential of this compound to be further developed for cancer treatment.

Unlike CQ and HCQ in which the autophagy inhibition and anti-tumor activity were observed when used at high concentrations^19–23^, ECDD-S27 exhibits a potent inhibition of the autophagic flux and cancer cell survival at low nanomolar concentrations. The IC_50_ values of ECDD-S27 for suppression of cell viability are 0.03–0.06 µM for cancer cells tested in our study while the IC_50_ values of HCQ are 11.44–18.40 µM, demonstrating that ECDD-S27 is >300 fold more potent than that of HCQ. Our results are consistent with other studies in which the IC_50_ values of HCQ in restricting various cancer cells were reported to be in the range of 8–72 µM^45^. In addition to their high concentration requirement, results from preclinical models and clinical trials have also demonstrated that while CQ and HCQ can be used to synergistically enhance the efficacy of other anti-cancer drugs and radiation, both have low efficacy when used as a single agent^15^. Due to the aforementioned limitations of CQ and HCQ and potent activity of ECDD-S27 as a single agent against cancer cells while sparing the normal cells, ECDD-S27 appears attractive for future therapeutic development. Therefore, studies to determine ECDD-S27 safety and efficacy in cancer animal models are warranted and require further investigation.

Although both CQ and HCQ have been shown to impair lysosomal acidification and hence the autophagic flux, their definitive molecular targets are unclear^46^. CQ and HCQ are lysomotropic weak base agents that accumulate in lysosomes resulting in the increased pH and diminished activity of the lysosomal enzymes^47^. However, protein or nonprotein targets of CQ and HCQ within the lysosomes remain to be identified. Better understanding of the underlying molecular mechanism elicited by CQ and HCQ and identifying their targets are keys to aid in improving their efficacy through structural modifications. In contrast, our work by the molecular docking, SERS, and functional study analyses identified the V-ATPase as the ECDD-S27 target.

The eukaryotic V-ATPase is a multi-subunit protein complex which functions as a motor for pumping H^+^ ^48^. It is composed of the peripheral catalytic V_1_ and membrane-bound V_0_ domains, in which the former functions in hydrolyzing ATP while the latter translocates the proton across membrane^49^. Our SERS analysis confirmed that ECDD-S27 can interact with the V-ATPase while our molecular docking analysis revealed that ECDD-S27 can form a stable interaction with the membrane-bound subunit a, c, c″, and e of the V_0_ domain. Particularly, ECDD-S27 forms a hydrogen bond interaction between its ether oxygen atom with the amide nitrogen atom of Gln55 on the subunit e. In addition, several hydrophobic interactions are formed between ECDD-S27 and amino acid residues within the subunit a, c, and c″. As previous studies have demonstrated the importance of subunit a, c, cʹ, and c″ in the proton transport and release into the lumen^50^, thus ECDD-S27 may inhibit the translocation of H^+^ and thereby inhibiting the lysosomal acidification. Indeed, our functional study confirmed this idea in which treatment of ECDD-S27 to different cancer cells demonstrated a dose-dependent decrease in the number of acidic organelles inside cells. Nevertheless, although the molecular docking, SERS, and functional studies strongly support that the V-ATPase is a target of ECDD-S27, additional target cannot be excluded and is thus a subject for future investigations.

## Methods

### Cells, inhibitors, antibodies, plasmid construct and fluorescent dye

Raw264.7, HT-29, HepG2, and HeLa cells were obtained from the American Type Culture Collection. Cells were maintained in DMEM (Gibco), 10% FBS (Gibco), 0.37% sodium bicarbonate (Sigma), and 4 mM *l*-glutamine (Hyclone) (full medium) at 37 °C and 5% CO_2_. Earle’s balanced salt solution (EBSS; starvation medium) and hydroxychloroquine sulfate (HCQ) were obtained from Sigma. Bafilomycin A1 (Baf; LC laboratories) was used at 0.1 µM. The plasmid constructs encoding RFP-GFP-LC3B and GFP-LC3B used in this study have been described previously^51^. For immunoblotting, polyclonal antibodies against p62 (Progen) were used at 1: 3,000 dilutions, polyclonal antibodies against LC3 (MBL International) were used at 1: 2,000 dilutions, polyclonal antibodies against V0a3 (Thermo Fisher) were used at 1: 2,000 dilutions, polyclonal antibodies against V1A1 (Thermo Fisher) were used at 1: 2,000 dilutions, and monoclonal antibody against Actin (Abcam) was used at 1: 10,000 dilutions. The fluorescent dye Hoechst 33342 (Invitrogen) was used at 1:500 and LysoTracker Red (LTR; Invitrogen) dye was used at 0.25 µM.

### Screening of natural product-derived compounds

Screening of natural product-derived compounds for their autophagy modulating activity was conducted by using the Operetta HC imaging analysis system (PerkinElmer) to quantitate the number of RFP-GFP-LC3B puncta (a cellular marker for autophagosomes/autolysosomes) in Raw264.7 macrophages. In brief, Raw264.7 macrophages were transfected with 5 µg cDNAs encoding RFP-GFP-LC3B in 100 µL of electroporation buffer V (Amaxa) using the Amaxa Nucleofector apparatus and the program D-032. The cells were then transferred to a new flask containing complete medium. At 24 h after transfection, 3 × 10^4^ cells were then plated into each well of 96-well plates. At 48 h after transfection, cells were treated with starvation (EBSS media; positive control; 10 wells per plate), DMSO (negative control; 10 wells per plate), or 50 µM final concentration of each natural product-derived compounds for 4 h. Cells were then fixed with 4% paraformaldehyde for 10 min and stained with Hoechst for 15 min. Cells were then analyzed by HC image analysis to quantify the number of fluorescent autophagic puncta, which are either RFP^+^GFP^+^-LC3B (autophagosomes) or RFP^+^GFP^−^LC3B (autolysosomes). Compounds that can up-regulate the number of autophagic puncta to be more than 3 S.D. above that of the mean of the negative control in the same plate were identified as positive compounds for autophagy inducers or autophagy flux inhibitors.

### ECDD-S27 synthesis and structure characterization

To synthesize ECDD-S27, cleistanthin A was first extracted from the aerial parts of *Phyllanthus taxodiifolius* using the previously described procedure^36^. All solvents used for extraction and isolation were distilled prior to use at their boiling point ranges. Cleistanthin A (306.5 mg, 0.568 mmol) was then reacted with 3,5-dimethoxybenzoic acid (155.1 mg, 0.852 mmol) in the presence of *N*,*N*′-dicyclohexylcarbodiimide (175.8 mg, 0.852 mmol) and 4-dimethylaminopyridine (cat.) at room temperature for 12 h. The reaction mixture was purified by column chromatography, eluting with 50% ethyl acetate-hexanes (1:1), followed by recrystallization from MeOH-CH_2_Cl_2_ to yield ECDD-S27 (**1**) (308.4 mg, 77% yield) as yellow powder. Purity of ECDD-S27 was determined to be 97% by using HPLC technique (Agilent 1200 series; column C_18_ shiseido, 80% MeOH/H_2_O, 1 mL/min). Melting points were measured using a digital Electrothermal melting point apparatus. Optical rotations were determined on JASCO DIP 370 digital polarimeter, using a 50 mm microcell (1 mL). UV (EtOH) and IR (KBr) spectra were recorded on a JASCO 530 and Perkin-Elmer spectrophotometers, respectively. The 400 MHz ^1^H and 100 MHz ^13^C NMR spectra were recorded on Bruker Ascend-400 spectrometer in CDCl_3_ using TMS as internal standard. EIMS were recorded at 70 eV (probe) on a Thermo Finnigan Polaris Q mass spectrometer. The HRMS were recorded on HR-TOF-MS (Micromass model VQ-Tof-2 spectrometer). Silica gel 60 (70−230 mesh ASTM) was used for column chromatography, and preparative TLC was carried out with silica gel 60 PF_254_ (5–40 μm, 0.5 mm). ECDD-S27 (**1**): m.p. 142.3–143.5 °C (MeOH-CH_2_Cl_2_); TLC (50% EtOAc-hexanes): R_f_ = 0.33; ${[\alpha ]}_{589}^{26.5}$ −42.6 (*c* 0.5 in CHCl_3_); UV (EtOH): λ_max_ (log ε): 206 (4.88), 261 (4.61), 308 (4.13), 355 (3.67) nm; CD (1.99 × 10^−5^ M, EtOH): λ_max_ (Δε): 314 (−2.58), 268 (+6.49), 250 (−2.94), 210 (+4.00), 200 (−0.82), 194 (+4.06); IR (KBr):3450, 2938, 1763, 1729, 1596, 1507, 1432, 1390, 1351, 1264, 1227, 1158, 1101, 1062, 1040, 931, 860, 770 cm^−1^; EI-MS *m/z* (rel. int.): 380 [M-Glucose]^+^ (25), 325 (6), 261 (28), 233 (6), 165 (100), 137 (14); HRMS (*m/z*): [M + Na]^+^ calcd. for C_37_H_36_O_14_Na, 727.2003; found, 727.2002; ^1^H NMR (400 MHz, CDCl_3_): $\delta$ 7.53 (d, J = 2.7 H, 1H), 7.22 (s, 2H), 7.02 (s, 1H), 6.94 (d, J = 7.9 Hz, 1 H), 6.80 (s, 1 H), 6.67 (s, 1 H), 6.09 (s, 1 H), 6.04 (s, 1 H), 5.58 (brt, J = 7.0 Hz, 1 H), 5.46 (d, J = 14.8 Hz, 1H), 5.39 (dd, J = 14.8, 2.7 Hz, 1 H), 5.19 (d, J = 6.6 Hz, 1 H), 4.21 (brdd, J = 11.8, 3.4 Hz, 1H), 3.87 (s, OCH_3_), 3.82 (s, 2 x OCH_3_), 3.77 (s, OCH_3_), 3.66 (s, OCH_3_), 3.58 (obsc, 1H), 3.56 (obsc, 1H), 3.55 (s, OCH_3_), 3.38 (m, 1H); ^13^C NMR (100 MHz, CDCl_3_): $\delta$ 169.6 (C), 165.3 (C), 160.8 (2 x C), 151.8 (C), 150.3 (C), 147.5 (2 x C), 144.0 (C), 135.8 (C), 131.3 (C), 130.6 (C), 128.4 (C), 127.3 (C), 126.4 (C), 123.6 (CH), 119.2 (C), 110.7 (CH), 108.2 (CH), 107.6 (2 x CH), 106.0 (CH), 105.6 (CH), 101.29 (CH), 101.23 (CH_2_), 100.8 (CH), 82.1 (CH), 78.3 (CH), 72.6 (CH), 67.1 (CH_2_), 63.2 (CH_2_), 60.4 (OCH_3_), 58.7 (OCH_3_), 56.0 (OCH_3_), 55.8 (OCH_3_), 55.6 (2 x OCH_3_).

### Immunofluorescence confocal microscopy and HC image analysis

Cells were transfected with 5 µg of cDNAs encoding RFP-GFP-LC3B or GFP-LC3B as described above. For confocal microscopy, at 24 h post transfection, cells were plated onto 12-well plate containing coverslips at a density of 3 × 10^5^ cells/well (Raw264.7 macrophages), 2.5 × 10^5^ cells/well (HT-29 or HepG2 cells), and 8 × 10^4^ cells/well (HeLa cells). At 48 h after transfection, cells were treated with DMSO (negative control), starvation (EBSS media; positive control), or ECDD-S27 at indicated final concentrations for 4 h. Cells were then fixed with 4% paraformaldehyde for 10 min and mounted with ProLong Gold antifade mountant (Invitrogen). The number of RFP^+^GFP^+^-LC3B or RFP^+^GFP^−^LC3B puncta per cell were quantified using Zeiss LSM-700 laser scanning confocal microscope. At least 30 cells per experimental condition in three independent experiments were analyzed. For HC image analysis of GFP-LC3B expressing cells, at 24 h post transfection, cells were plated onto 96-well black plate at a density of 1 × 10^4^ cells/well (HT-29 or HepG2 cells) and 2 × 10^3^ cells/well (HeLa cells). At 48 h after transfection, cells were treated with DMSO (negative control) or ECDD-S27 at indicated final concentrations for 72 h. Cells were then fixed with 4% paraformaldehyde for 10 min and stained with Hoechst for 15 min. Percent cell viability and autophagic flux-inhibited cells (defined as cells with more than 5 GFP^+^-LC3B puncta) were analyzed by HC image analysis.

### SDS-PAGE and immunoblotting

Cells were lysed in lysis buffer containing 62.5 mM Tris-HCl (pH 6.8), 10% glycerol, 2% SDS, 5% β-mercaptoethanol, and 0.01% bromophenol blue. The cell lysates were then separated by 15% polyacrylamide gels and transferred onto nitrocellulose membranes (Amersham Biosciences). The membranes were then blocked with 5% blocking solution (Roche Diagnostics) for 1 h at room temperature followed by incubation with primary antibodies at 4 °C overnight. The membranes were washed 4 times with 0.1% PBST and incubated with appropriate horseradish peroxidase-conjugated secondary antibodies for 1 h at room temperature. The membranes were then washed 4 times and the expression levels of the proteins were detected using the chemiluminescence method (Roche Diagnostics). The band intensities were quantified using the Image J 1.47 v software (NIH, USA). At least three independent experiments were performed and analyzed. IC_50_ values were the concentrations at which 50% increase in the p62/Actin or LC3II/Actin levels could be observed, relative to that seen in the bafilomycin A1 treated control set to 100%.

### MTS cell viability assay

HT-29 (1 × 10^4^ cells), HepG2 (1 × 10^4^ cells), and HeLa (2 × 10^3^ cells) were plated onto 96-well plates for 16 h. Cells were treated with DMSO (negative control), ECDD-S27, HCQ, or bafilomycin A1 at the indicated concentrations and incubated for 72 h. The cell viability was measured by MTS assay using CellTiter 96 AQueous One Solution Reagent (Promega) following the manufacturer’s instruction. The percent cell viability was then calculated by using % cell viability = [(Absorbance of treated cells − Absorbance of blank)/(Absorbance of DMSO control cells − Absorbance of blank)] × 100. The percent cell cytotoxicity was calculated by using % cell cytotoxicity = 100 − % cell viability. IC_50_ values were then determined by plotting the dose response curve between Log [M] concentrations of the compound and % cell cytotoxicity and the values were determined using nonlinear regression analysis by GraphPad Prism 5 (GraphPad Software Inc.). All assays were performed in triplicate with at least three independent experiments.

### Molecular simulation

The X-ray crystal structure of the eukaryotic V-ATPase (PDB id 5TJ5)^52^ from *Saccharomyces cerevisiae* was used as the docking target. Prior to the docking simulation, the protein structure was subjected to molecular dynamics simulation. Briefly, molecular dynamics simulation was performed using the AMBER14 force field^53^ in YASARA, version 18.4.24^54^. The simulation was performed using default parameters in *md_runmembrane.mcr* for protein-lipid membrane system in YASARA. Briefly, the protein was placed in a lipid membrane (i.e. phosphatidyl-ethanolamine) corresponding to the XZ plane in which the membrane spans 15 Å on each side of the protein. This lipid-protein system is immersed in water that spans 10 Å on each side of the protein. The simulation was performed for 30 ns at pH 7.4, temperature of 298 K and 0.9% NaCl solution.

Docking calculation was performed using AutoDock, version 4.2^55^, by means of the Lamarckian Genetic Algorithm^56^ while the grid box was generated using AutoGrid^57^. The protein structure was prepared for docking by first adding essential hydrogen atoms, Kollman united atom charges and solvation parameters using AutoDockTools^55^ and PyRx0.3^58^. The ligand structure was geometrically optimized via molecular mechanic force field using the Python package, RDKit. Next, non-polar hydrogen atoms were merged, Gasteiger partial charges were added and rotatable bonds were defined. As there was no reported information on binding site residues, therefore a box was generated to cover all residues of the protein structure. AutoDock parameters pertaining to set- and distance-dependent dielectric functions were used for calculating van der Waals and electrostatic terms, respectively. The initial position, orientation and torsions of the ligand was set randomly. The docked compound was derived from 100 independent docking runs using a mutation rate of 0.02 and a crossover rate of 0.8 and the calculations were set to terminate after a maximum of 2.5 × 10^6^ energy evaluations were reached. Furthermore, the population size was set to use 250 randomly placed individual. The Lamarckian genetic algorithm was employed to search for low-energy binding orientations. A translational step of 0.2 Å, a quaternion step of 5 Å and a torsion step of 5 Å were used. The best docked conformations as deduced from the clustering histogram were those with low binding energy.

Docking results were analyzed using AutoDockTools^55^ and PyMOL^59^. These tools help to shed light on the interaction type (i.e. hydrogen-bond, π-π interaction and cation-pi interaction) contributing to the ligand binding. Favorable ligand binding poses (i.e. deduced by clustering histograms) along with their corresponding binding energy were obtained from AutoDockTools^55^. Moreover, PyMOL^59^ was used to provide complementary information on ligand-receptor interaction. All molecular graphics were rendered and ray-traced using PyMOL^59^.

### Immunoprecipitation and surface-enhanced Raman substrates (SERS) analysis

HeLa cells were lysed in RIPA buffer supplemented with the protease inhibitor cocktail tablet (Roche) on ice for 20 min. The lysate was then pass through 27 gauge needle 9 times and then spun down at 10,000 × g at 4 °C for 10 min. The supernatant was transferred to a new tube. Protein concentration was then determined using RC-DC kit (Biorad) and 2.5 mg of total lysate was aliquoted into new tubes. Rabbit anti-V1A1 antibodies were added to the lysate at 2 µg per mg protein using normal rabbit IgG (Santa Cruz Biotechnology) as the control. The samples were then incubated at 4 °C for 4 h. Protein A sepharose beads (Santa Cruz Biotechnology) were then added and incubated at 4 °C for 4 h. The immunoprecipitated proteins were then washed with PBS containing 0.5% NP-40 three times. The immune complex was then subjected for WB analysis to check for the presence of V1A1 and V0a3 proteins in the immune complex as described above.

For SERS analysis, the anti-V1A1 precipitated immune complex was treated with 5 mM ECDD-S27 at 4 °C overnight. The sample was then washed three times in 1 mL PBS containing 0.5% NP-40 in order to wash out the unbound ECDD-S27. The washed sample was then spotted onto the surface-enhanced Raman substrates (SERS) ONSPEC Chips (NECTEC, Thailand), which is fabricated in silver nanorod structure. All the SERS experiments were performed with the Raman spectrometer (Rigaku Analytical Devices, Inc.). As the controls, the ECDD-S27 alone, buffer alone, and anti-V1A1 immune complex alone were spotted onto the ONSPEC Chips followed by the Raman analysis. All spectra were taken in an automatic mode by the Raman spectroscope with 1064 nm laser wavelength and TE cooled InGaAs 512 pixel detector. The range of spectrum cover 200–2500 ${{cm}}^{-1}$ with resolution 8–11 ${{cm}}^{-1}$ and adjustable power laser 30–490 mW.

### LysoTracker Red staining

HT-29 (2.5 × 10^4^ cells), HepG2 (2.5 × 10^4^ cells), or HeLa (6 × 10^3^ cells) were plated onto 96-well black plates for 16 h. Cells were then treated with DMSO (negative control) or ECDD-S27 at the indicated concentrations for 2 h. LysoTracker Red (LTR) dye was then added and the incubation was continued for 2 h. Cells were then fixed with 4% paraformaldehyde for 10 min and stained with Hoechst for 15 min. The LTR^+^ puncta/cell were analyzed by HC image analysis. At least 30 cells per experimental condition were analyzed.

### Statistical analysis

Unless otherwise stated, all experiments were conducted at least three times and the data were pooled for determination of the mean ± standard error of the mean (S.E.M.). All data were analyzed by the Prism software (GraphPad) using one-way ANOVA with a Tukey’s multiple comparison test. *p* values less than 0.05 were considered to indicate statistical significance.

## Supplementary information

Supplementary Information

## Data Availability

The datasets generated during and/or analysed during the current study are available from the corresponding author on reasonable request.
