# Supplementary material for: A novel potent autophagy inhibitor ECDD-S27 targets vacuolar ATPase and inhibits cancer cell survival
Source: Sci Rep. 2019 Jun 24;9:9177. doi: 10.1038/s41598-019-45641-x (PMC6591302; doi:10.1038/s41598-019-45641-x)

Supplementary Information

A novel potent autophagy inhibitor ECDD-S27 targets vacuolar ATPase and inhibits cancer cell survival

Jiraporn Paha[1], Phongthon Kanjanasirirat[2], Bamroong Munyoo[2,3], Patoomratana Tuchinda[2,3], Naravut Suvannang[2], Chanin Nantasenamat[4], Kanokthip Boonyarattanakalin[5], Prasat Kittakoop[6,7,8], Sirawit Srikor[1], Gunganist Kongklad[9], Noppawan Rangkasenee[2], Suradej Hongeng[2,10], Pongsak Utaisincharoen[1], Suparerk Borwornpinyo[2,11] & Marisa Ponpuak[1,*]

Supplementary Methods

Cell toxicity assay

Human kidney normal cell HK-2 cells were obtained from ATCC. Cells were maintained in DMEM (Dulbecco's Modified Eagle Medium) supplemented with 10% FBS, 1% penicillin/streptomycin, and 1% HEPES at 37°C in 5% CO_2. At 24 h before treatment, HK-2 cells (1 x 10^4 cells) were plated onto 96-well plates. Cells were then treated with DMSO (negative control) or different concentrations of ECDD-S27 for 72 h. Cell toxicity was then measured by using the MTT assay (Sigma-Aldrich) per manufacturer's recommendation. The absorbance at 570 nm was determined by Multi-Mode Microplate Reader (ENVISION, Perkin Elmer).

Supplementary Figures

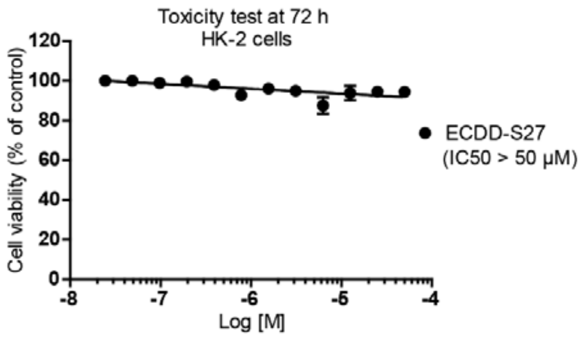

Supplementary Fig. S1. ECDD-S27 is not toxic to human kidney normal HK-2 cells. HK-2 cells were treated with DMSO (negative control) or varied concentrations of ECDD-S27 for 72 h and cell toxicity was measured by the MTT assay. Data are mean ± SEM done in triplicate; the results were expressed relative to the DMSO control, defined as 100%.

Images of immunoblotting

Figure 1e

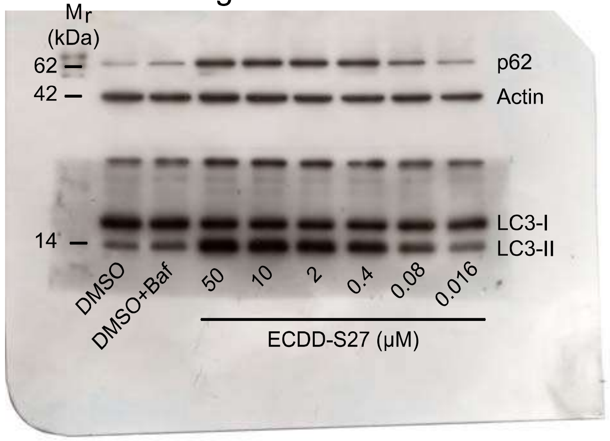

Figure 3a

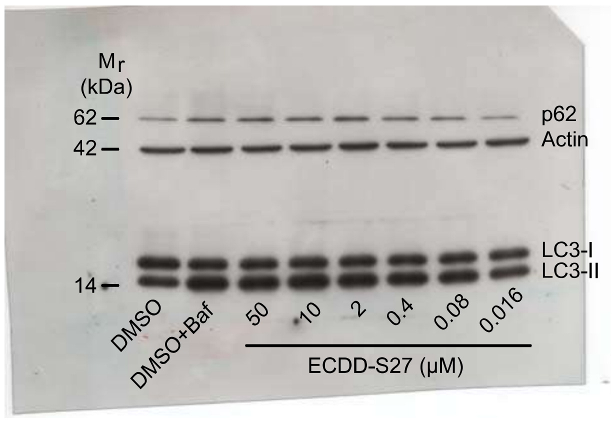

Figure 4a

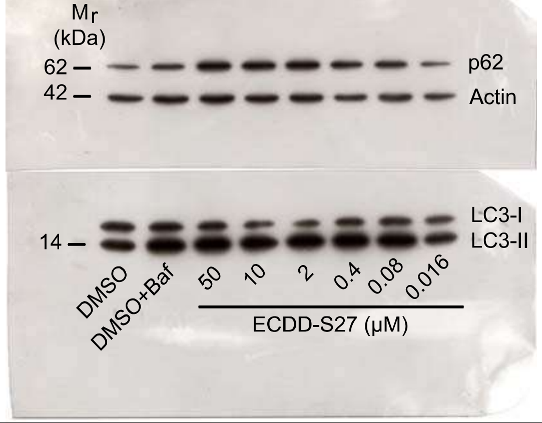

Figure 5a

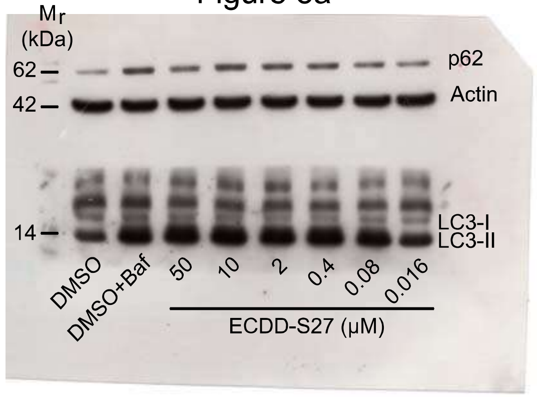

Figure 7a

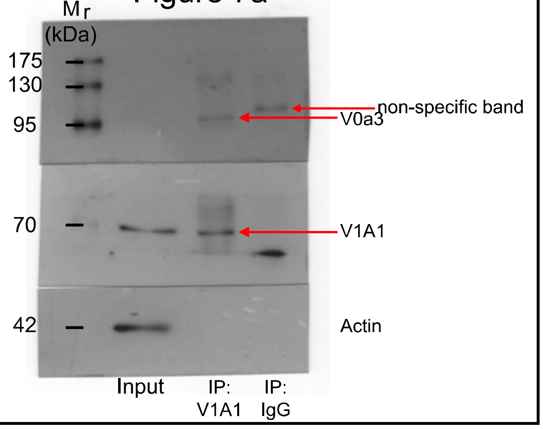

Supplement: Supplementary file 1 — Supplementary Information [file 41598_2019_45641_MOESM1_ESM.pdf]
